# Pericytes/vessel-associated mural cells (VAMCs) are the major source of key epithelial-mesenchymal transition (EMT) factors SLUG and TWIST in human glioma

**DOI:** 10.18632/oncotarget.25275

**Published:** 2018-05-08

**Authors:** Lisa Mäder, Anna E. Blank, David Capper, Janina Jansong, Peter Baumgarten, Naita M. Wirsik, Cornelia Zachskorn, Jakob Ehlers, Michael Seifert, Barbara Klink, Stefan Liebner, Simone Niclou, Ulrike Naumann, Patrick N. Harter, Michel Mittelbronn

**Affiliations:** ^1^ Edinger Institute (Neurological Institute), Goethe University, Frankfurt, Germany; ^2^ Department of Neuropathology, University of Heidelberg, Heidelberg, Germany; ^3^ Clinical Cooperation Unit Neuropathology, German Cancer Research Center (DKFZ), Heidelberg, Germany; ^4^ Charité — Universitätsmedizin Berlin, Corporate Member of Freie Universität Berlin, Humboldt-Universität zu Berlin, and Berlin Institute of Health, Department of Neuropathology, Berlin, Germany; ^5^ Laboratory of Molecular Neuro-Oncology, Department of Vascular Neurology, Hertie Institute for Clinical Brain Research, University of Tübingen, Tübingen, Germany; ^6^ German Cancer Consortium (DKTK), Heidelberg, Germany; ^7^ German Cancer Research Center (DKFZ), Heidelberg, Germany; ^8^ Department of Radiation Oncology, University of Tübingen, Tübingen, Germany; ^9^ Carl Gustav Carus Faculty of Medicine, Technische Universität Dresden, Institute for Medical Informatics and Biometry (IMB), Dresden, Germany; ^10^ National Center for Tumor Diseases (NCT), Dresden, Germany; ^11^ Institute for Clinical Genetics, Faculty of Medicine Carl Gustav Carus, Dresden University of Technology, Dresden, Germany; ^12^ NORLUX Neuro-Oncology Laboratory, Luxembourg Institute of Health (LIH), Strassen, Luxembourg; ^13^ Luxembourg Centre of Neuropathology (LCNP), Dudelange, Luxembourg; ^14^ Laboratoire National de Santé (LNS), Dudelange, Luxembourg; ^15^ Luxembourg Centre for Systems Biomedicine (LCSB), University of Luxembourg, Esch-sur-Alzette, Luxembourg

**Keywords:** EMT, MET, gliomas, pericytes, vessel-associated mural cells

## Abstract

Epithelial-to-mesenchymal transition (EMT) is supposed to be responsible for increased invasion and metastases in epithelial cancer cells. The activation of EMT genes has further been proposed to be important in the process of malignant transformation of primary CNS tumors. Since the cellular source and clinical impact of EMT factors in primary CNS tumors still remain unclear, we aimed at deciphering their distribution *in vivo* and clinico-pathological relevance in human gliomas.

We investigated 350 glioma patients for the expression of the key EMT factors SLUG and TWIST by immunohistochemistry and immunofluorescence related to morpho-genetic alterations such as *EGFR*-amplification, *IDH-1* (R132H) mutation and 1p/19q LOH. Furthermore, transcriptional cluster and survival analyses were performed.

Our data illustrate that SLUG and TWIST are overexpressed in gliomas showing vascular proliferation such as pilocytic astrocytomas and glioblastomas. EMT factors are exclusively expressed by non-neoplastic pericytes/vessel-associated mural cells (VAMCs). They are not associated with patient survival but correlate with pericytic/VAMC genes in glioblastoma cluster analysis.

In summary, the upregulation of EMT genes in pilocytic astrocytomas and glioblastomas reflects the level of activation of pericytes/VAMCs in newly formed blood vessels. Our results underscore that the negative prognostic potential of the EMT signature in the group of diffuse gliomas of WHO grade II-IV does most likely not derive from glioma cells but rather reflects the degree of proliferating mural cells thereby constituting a potential target for future alternative treatment approaches.

## INTRODUCTION

The concept of epithelial-to-mesenchymal transition (EMT) was first reported over 40 years ago [1]. EMT is a well-controlled mechanism in development, however increasing data indicate its reactivation in cancer [2]. The reactivation of EMT genes is associated with worse patient prognosis in carcinomas [3–6]. In contrast, neuroectodermal tumors have only been poorly investigated for EMT factors so far. In distinct molecular subclasses in high-grade gliomas a so-called mesenchymal signature is related to poor prognosis [7]. Recently, *in vitro* and animal studies provided evidence that the EMT program might play a role in CNS neoplasms since over-expression of EMT regulator molecules let to enhanced glioma cell invasion [8]. Importantly, gene expression datasets revealed a shorter time to recurrence in patients showing an activation of EMT genes [9]. Additionally, a correlation between the expression of so-called “glioma stem cell” markers with EMT regulators has been demonstrated [10]. Meanwhile it is also accepted that this process is revertable and referred to mesenchymal-to-epithelial transition (MET) [1, 11]. Therefore, MET induction has been proposed as a promising therapeutic strategy for glioblastomas by potentially reversing the malignancy-associated EMT program thereby reducing glioma cell invasion as well as the survival of glioma-initiating cells. However, analyses of different glioma grades also indicated an association of mesenchymal markers not only with malignant high-grade gliomas but also with the low-grade slowly growing pilocytic astrocytoma [12]. Of note, most studies linking the expression of EMT genes to glioma malignancy analyzed gene expression at mRNA levels but not the related protein expression *in vivo* [13]. Nevertheless, the EMT signature in glioblastoma was primarily ascribed to glioma cells without deciphering the exact cellular sources of the expression of EMT transcription factors. Therefore, the aim of our study was to dissect the cellular source of expression of key EMT factors and investigate their potential association with patient survival in human gliomas. Since many pathways, among others including the WNT, RTK, TGFβ pathways, are involved in the EMT program, we mainly focused on down-stream EMT transcription factors such as SLUG and TWIST.

## RESULTS

### EMT molecules are exclusively expressed in pilocytic astrocytomas and glioblastomas amongst human astrocytomas

**Figure 1:**
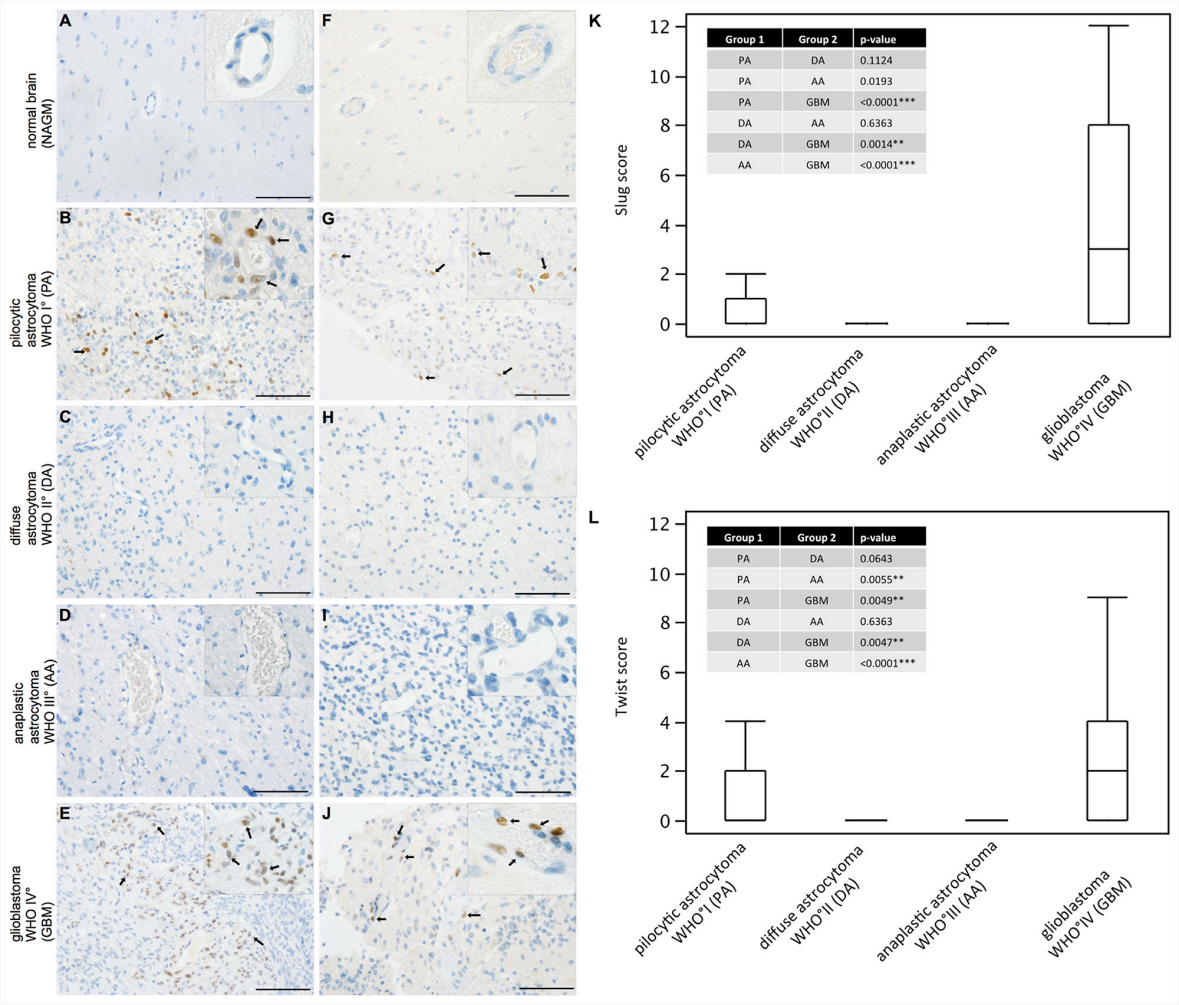
SLUG and TWIST are expressed in pilocytic astrocytomas and glioblastomas in human astrocytic tumors Immunohistochemical analyses showing SLUG (*left column)* and TWIST (*right column)* expression (SLUG and TWIST positive nuclei indicated with black arrows) in **(A, F)** normal appearing grey matter (NAGM), **(B, G)** pilocyctic astrocytoma WHO grade I showing vascular proliferations, **(C, H)** diffuse astrocytoma WHO grade II, **(D, I)** anaplastic astocytoma WHO grade III and **(E, J)** glioblastoma WHO grade IV showing vascular proliferations. (Scale bar = 100μm). Box-and-Whisker plots for **(K)** SLUG and **(L)** TWIST expression scores are depicted. P-values from all pair comparisons of different WHO grade astrocytomas were obtained using the non-parametric Wilcoxon test followed by Bonferroni post hoc test (p < 0.05 = ^*^; p < 0.01 = ^**^; p < 0.0001 = ^***^). Pilocytic astrocytoma, WHO grade I (PA; n=47); diffuse astrocytoma WHO grade II (DA; n=16); anaplastic astrocytoma WHO grade III (AA; n=35); glioblastoma WHO grade IV (GBM; n=252).

While SLUG expression was absent in normal human grey (Figure 1A) and white matter, slowly growing pilocytic astrocytomas WHO grade I (Figure 1B) partly showed prominent SLUG expression in a perivascular distribution. In contrast, both WHO grade II (Figure 1C) and WHO grade III (Figure 1D) astrocytomas were virtually devoid of SLUG expression. In glioblastomas WHO grade IV (Figure 1E) a similar SLUG distribution pattern as in pilocytic astrocytomas was observed. Similarly, TWIST was absent in normal grey (Figure 1F) and white matter while vessel-associated TWIST expression was seen in pilocytic astrocytomas (Figure 1G). Both diffuse (Figure 1H) and anaplastic (Figure 1I) astrocytomas remained TWIST-negative, but prominent TWIST expression was focally observed in glioblastomas (Figure 1J) in a perivascular localization. SLUG (Figure 1K) and TWIST (Figure 1L) expression was significantly higher in glioblastoma as compared to all other astrocytomas. Pilocytic astrocytomas showed second highest SLUG and TWIST expression scores in astrocytoma subgroups (Table 1) reaching significance in comparison to WHO grade III astrocytomas for TWIST expression. We also investigated ZEB-1 expression in our cohort, since ZEB-1 is considered an important factor in the activation process of the EMT program. However, we found that ZEB-1 was strongly expressed in endothelial, pericytic as well as tumor cells in all WHO grades (data not shown). Therefore, we did not further proceed with the analysis of ZEB-1 in the present study.

**Table 1:** Frequencies of scores

	Score 0	Score 1	Score 2	Score 3	Score 4	Score 6	Score 8	Score 9	Score 12
PA°I – SLUG	32	4	3	0	2	2	0	2	0
DA°II – SLUG	7	0	0	0	0	0	0	0	0
AA°III – SLUG	22	0	1	0	0	0	0	0	0
GBM°IV – SLUG	76	22	23	5	25	31	43	11	12
PA°I – TWIST	28	1	7	0	2	0	2	2	0
DA°II – TWIST	7	0	0	0	0	0	0	0	0
AA°III – TWIST	22	0	0	0	1	0	0	0	0
GBM°IV – TWIST	98	19	26	8	36	26	22	4	8

PA°I = pilocytic astrocytoma, WHO grade I; DA°II = diffuse astrocytoma, WHO grade II; AA°III = anaplastic astrocytoma, WHO grade III; GBM°IV = glioblastoma, WHO grade IV

### SLUG and TWIST are expressed by non-neoplastic, blood vessel associated cells in human astrocytomas

**Figure 2:**
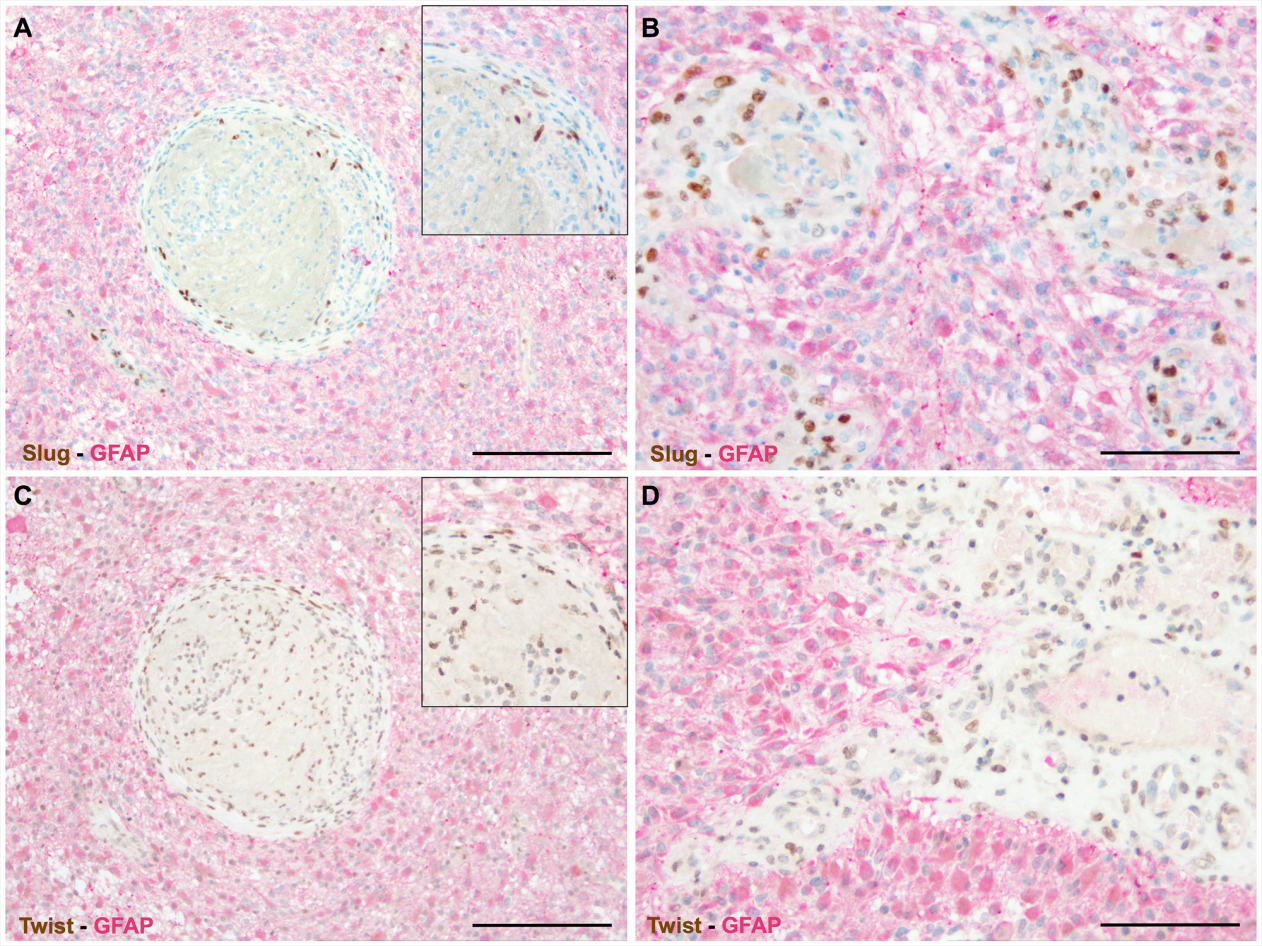
EMT factors are exclusively restricted to vascular proliferations in human astrocytomas Double immunohistochemistry for GFAP (*red*) and **(A, B)** SLUG (*brown*) as well as **(C, D)** TWIST (*brown*) indicating that all cells expressing the EMT transcriptions factors are GFAP-negative and are located within abnormal vascular complexes. (insets: higher magnifications; scale bar in A, C = 200μm and in B, D = 100μm).

**Figure 3:**
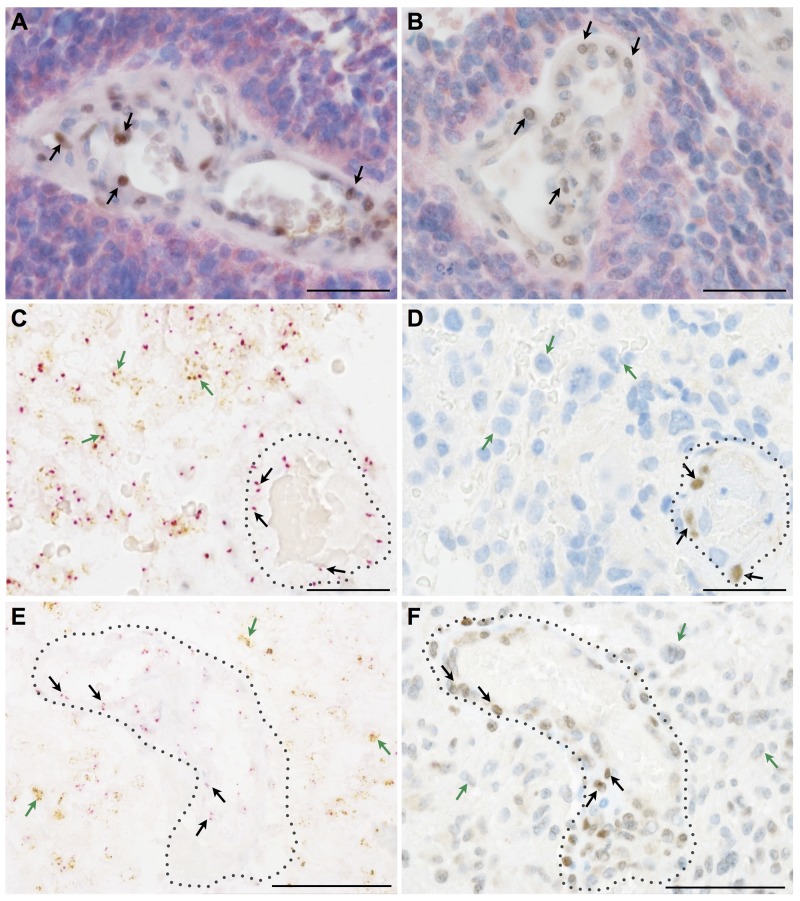
Key EMT molecules are expressed by non-neoplastic vessel-associated cells in human astrocytomas **(A, B)** Double immunohistochemistry for mutated *IDH-1* (R132H) (*red*) as a marker for neoplastic glial cells in a secondary glioblastoma together with (A) SLUG (*brown, arrows*) or (B) TWIST (*brown, arrows*) indicating that EMT molecules are restricted to *IDH-1* (R132H)-negative, vessel-associated cells. **(C, E)** Silver in-situ hybridization (SISH) staining (*brown dots; green arrows*) for *EGFR*-amplification indicating glial cells in primary glioblastoma and chromosome 7 control probes (*red dots; red arrows*) combined with **(D)** SLUG and **(F)** TWIST immunohistochemistry showing that mutated glioma cells are negative for EMT molecules, the latter being present on tumor-associated blood vessels (dotted lines; scale bars: A-F 50μm).

We first excluded by double immunohistochemistry that GFAP (glial fibrillary acidic protein) positive cells were the cellular source of SLUG (Figure 2A, 2B) and TWIST (Figure 2C, 2D). Next, we made use of morpho-genetic methods to unequivocally detect mutated proteins or amplified genes. In secondary glioblastomas *IDH-1* (R132H) mutated glioma cells were completely devoid of the EMT transcription factors SLUG (Figure 3A) and TWIST (Figure 3B) while vascular proliferations were constantly positive for those factors. Of note, SLUG (median test: p=0.0098) and TWIST (median test: p=0.0315) expression were significantly higher in *IDH-1*-wildtype as compared to *IDH-1*-mutant (R132H) glioblastomas, most probably related to the a priori more prominent neoangiogenesis in primary glioblastoma (data not shown). In contrast, *IDH-1*-mutant (R132H) glioblastomas mainly present as secondary glioblastoma deriving from lower grade gliomas that – per definition – do not display vascular proliferations. In lower grade *IDH-1* (R132H) mutated astrocytomas without signs of neo-angiogenesis, SLUG (Supplementary Figure 1A) and TWIST (Supplementary Figure 1B) were absent on both *IDH-1* (R132H)-mutated glioma cells and tumor-associated blood vessels. Furthermore, *EGFR*-amplified glioblastoma cells (Figure 3C, 3E) lacked co-expression with the EMT molecules SLUG (Figure 3D) or TWIST (Figure 3F). In contrast, in tumor-associated perivascular areas without amplified *EGFR* strong SLUG (Figure 3D) and TWIST (Figure 3F) expression was detected. Finally, in oligodendrogliomas with 1p/19q LOH, SLUG- and TWIST-positive cells were only encountered in perivascular cells displaying 2 copies for both the short as well as the long arm on chromosomes 1 and 19 (data not shown). Of note, also vascular or pericytic tumors (n=2 for each entity) displayed SLUG and TWIST expression (Supplementary Figure 2). While slowly growing hemangioblastoma only displayed single SLUG- or TWIST-positive cells in close proximity to blood vessels (Supplementary Figure 2A, 2B), the more malignant angiosarcomas displayed considerably more SLUG- and TWIST-positive cells located at similar localization as seen in pilocytic astrocytoma and glioblastoma (Supplementary Figure 2C, 2D). The most prominent SLUG- and TWIST-staining was encountered in hemangiopericytomas, a tumor considered to be of pericytic origin (Supplementary Figure 2E, 2F). This data indicates that SLUG and TWIST expression might be generally important for the activation or even malignant transformation of vessel-associated mural cells or pericytes.

### Pericytes/vascular associated mural cells (VAMCs) are the source of EMT factors in astrocytomas

**Figure 4:**
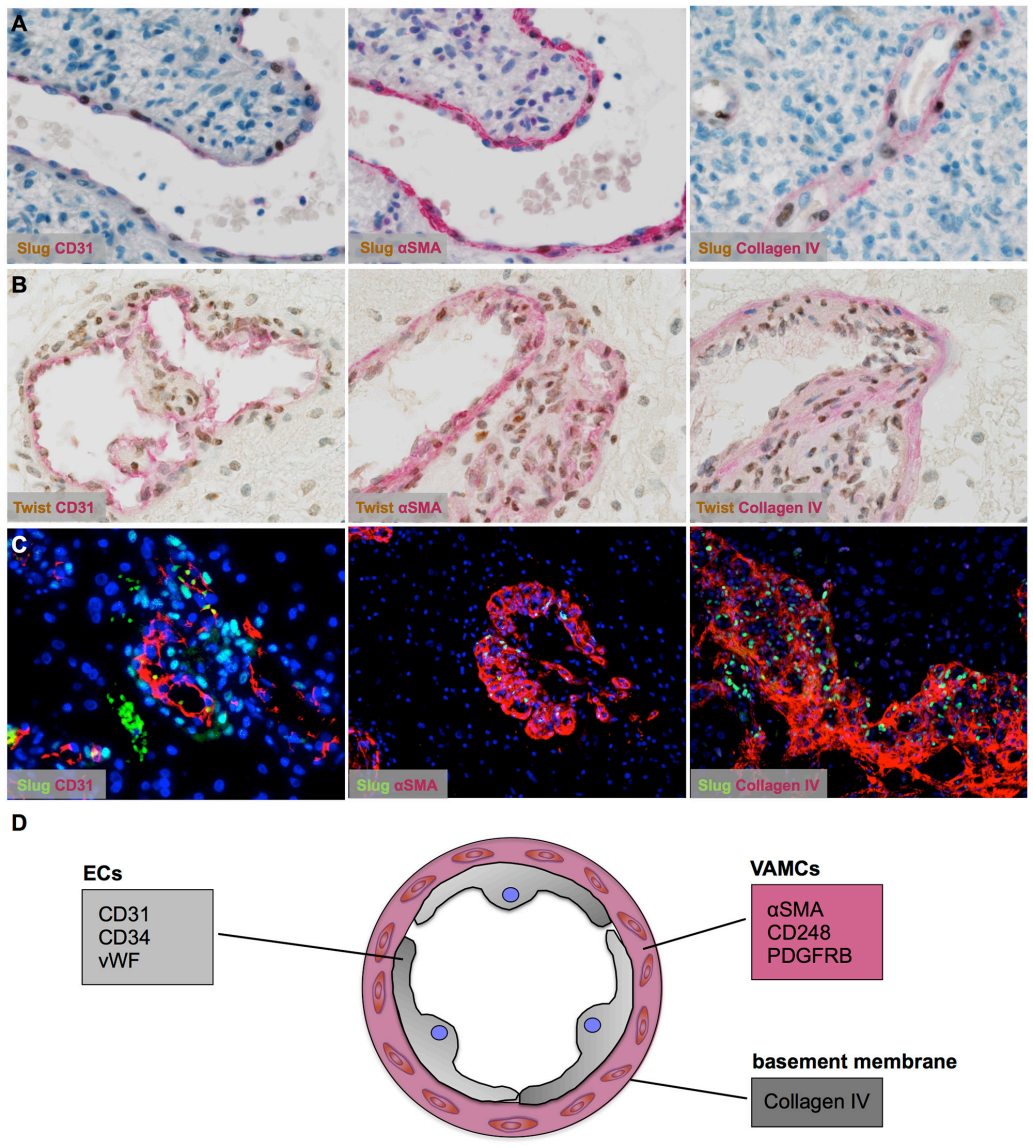
Vascular-associated mural cells (VAMCs)/pericytes are the source of SLUG and TWIST in human astrocytomas **(A, B)** Double immunhistochemistry for markers (*red*) of the different vascular layers such as endothelial cells (*left*; CD31), VAMCs/pericytes (*middle*; αSMA) and the basement membrane (*right*; collagen IV) in combination with (A) SLUG (*brown*) and (B) TWIST indicating EMT factor expression restricted to VAMCs/pericytes in human glioblastomas. **(C)** Immunofluorescence staining of SLUG (*green*) and CD31 (*left*), αSMA (*middle*) and collagen IV (*right, all coloured in red*) in human glioblastoma. A DAPI/ To-Pro 3 mix was used for nuclear counterstain (*blue*). **(D)** Graphical scheme of the different markers for distinct vascular layers: epithelial cells (ECs) and vascular associated mural cells (VAMCs) surrounded by basement membrane constituents.

After deciphering non-neoplastic cells of the vascular compartment as source of EMT factors in astrocytomas, we aimed to define the exact cell type of SLUG and TWIST expression. SLUG (Figure 4A; Supplementary Figure 3A, 3C) and TWIST (Figure 4B; Supplementary Figure 3B, 3D) were almost exclusively expressed by αSMA-positive VAMCs strictly located within the vascular compartment delineated by basement membrane constituents such as collagen IV (Figure 4C). In contrast, CD31-positive endothelial cells remained negative for EMT molecules SLUG and TWIST (for summary see Figure 4D).

### Gene expression of EMT transcription factors cluster with VAMC markers in TCGA glioblastoma samples

**Figure 5:**
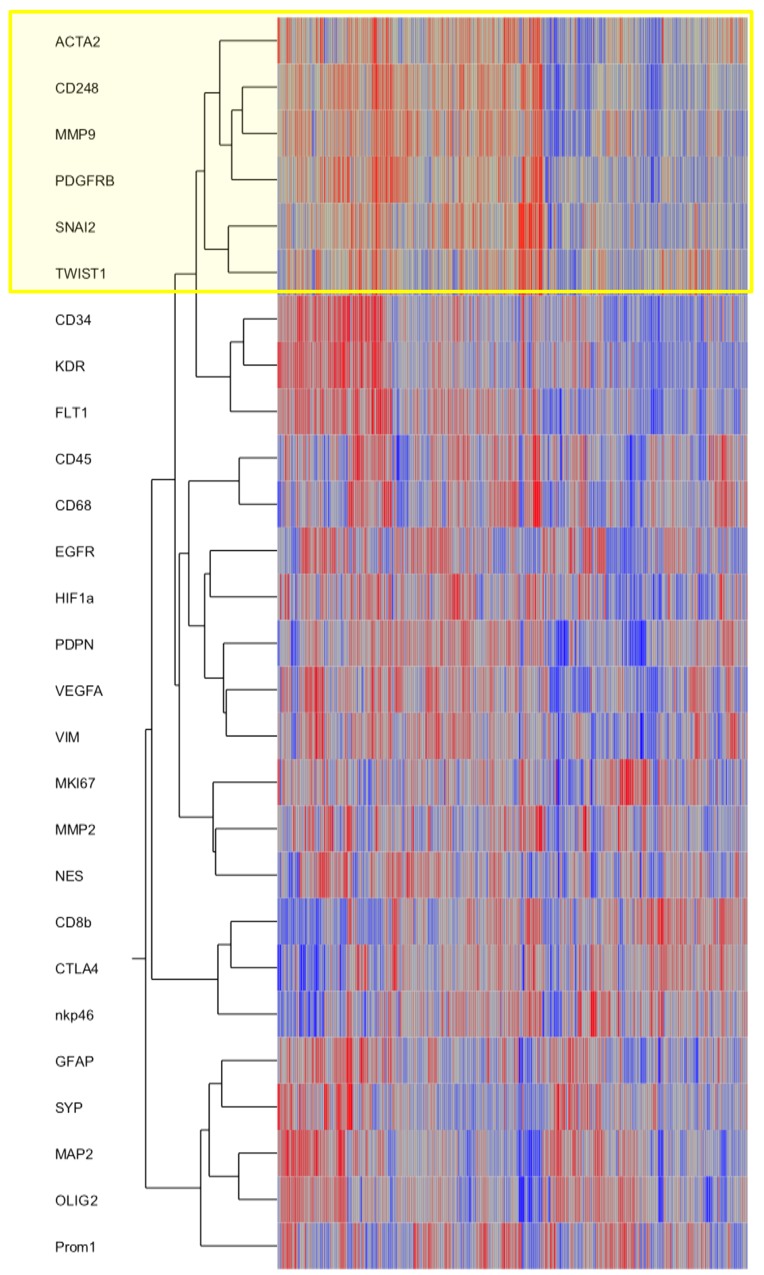
SNAI2 and TWIST mRNA expression clusters with VAMCs/pericytic markers Hierarchical cluster analyses of gene expression signatures of selected key factors of EMT, glioblastoma cells as well as glioblastoma micromilieu of 424 primary glioblastomas and 11 normal brain samples deriving from the TCGA data portal using the Agilent 244K G4502A microarray to determine mRNA profiles. Compared marker profiles: ACTA2, CD248, PDGFRB (pericytes/ VAMCs), CD34, KDR, FLT1 (endothelial cells), CD45 (leukocytes), CD68 (microglia/macrophages), lCD8b, CTLA4, nkp46 (lymphocytes), GFAP, MAP2, OLIG2, PROM1, NES, EGFR, VIM (distinct differentiation of glioma cells), SYP (neurons), HIF1a, VEGFA (hypoxia, angiogenesis) or MMP2, MMP9 (migration). Hierarchical clustering of this data was performed using the Ward’s minimum variance method.

To strengthen our *in vivo* findings, we assessed the TCGA database (Figure 5). We included key genes involved in the EMT process such as *SNAI2* (gene encoding SLUG protein) and *TWIST* in a cluster analysis together with selected markers for pericytes/VAMCs, endothelial and immune cells as well as resident or neoplastic neuroectodermal cells. Furthermore, central factors of hypoxia, angiogenesis or cell migration/invastion were included. The hierarchical clustering revealed that EMT genes (SNAI2, TWIST) cluster with pericytic/VAMC markers (ACTA2, CD248, PDGFRβ indicating a strong correlation and spatial association of EMT factors and pericytic/VAMC genes (Figure 5 and Supplementary Figure 4).

### EMT transcription factors are not associated with patient survival in glioblastomas

Since the activation of the EMT program has been proposed as a marker for glioma progression and worse patient prognosis, we analysed the association of EMT factor expression with patient survival in a large cohort of primary glioblastomas. Neither high SLUG (Supplementary Figure 5A) nor TWIST (Supplementary Figure 5B) were significantly associated with a worse patient survival. In contrast, patients with higher TWIST levels displayed longer survival rates almost reaching level of significance (p=0.05).

## DISCUSSION

Our results show that key EMT factors such as SLUG and TWIST are overexpressed in glioblastomas (Figure 1). Surprisingly, also benign, non-diffusely infiltrating pilocytic astrocytomas, WHO grade I, exhibit prominent expression of EMT molecules while WHO grade II or III astrocytomas are devoid of SLUG and TWIST. This is of interest, since EMT was proposed as a general mechanism in glioma progression and malignancy [7, 9]. This assumption was corroborated by *in vitro* studies showing that SNAIL or TWIST modulated glioma cell proliferation, migration and invasion [8, 14]. Experimentally, EMT in glioma cells is either triggered by hypoxia and consecutive upregulation of the vascular endothelial growth factor (VEGF) or a hypoxia-independent induction via hepatocyte growth factor (HGF)-mediated MET phosphorylation. Both events lead to a pro-invasive glioma cell phenotype [15, 16, 17]. In contrast to the hypothesis that the activation of EMT genes might also be responsible for the malignant transformation of glioma cells, our results indicate that the central EMT regulators SLUG and TWIST are exclusively expressed by pericytes/VAMCs of proliferating blood vessels (Figures 1–4) while being absent from glioma cells (Figures 2, 3). This is further strengthened by our findings that also vascular tumors such as hemangioblastomas and angiosarcomas display SLUG and TWIST expression in similar cellular association as seen in gliomas, while in hemangiopericytoma – a tumor considered to be of pericytic origin – the majority of tumor cells were moderately to strongly SLUG- and TWIST-positive (Supplementary Figure 2). Previous studies already hypothesized that the expression of EMT factors could be linked to tumour-associated stromal cells [18]. Although appearing contradictory to findings at mRNA levels at first glance [7, 9], a closer look at the data shows that our findings are in line with those studies. In our study, the term pericytes/VAMCs was chosen since it is very difficult to exactly define the functional status of the respective cells apart from their expression of pericytic markers. A re-analysis of datasets of pioneering mRNA signature studies revealed that classical primary glioblastomas with gains of chromosome 7 and losses of chromosome 10 were associated with necrosis. Most cases without necrotic transformation exhibited no large genetic alteration of both chromosomes indicating that those cases most likely were lower grade astrocytomas or secondary glioblastomas [7, 9]. After stratification of diffuse astrocytomas into subtypes with mesenchymal, proneural and proliferative molecular signature, it became evident that almost all WHO grade III astrocytomas cluster within the proneural signature while both glioblastomas, WHO grade IV, and pilocytic astrocytomas, WHO grade I, are much more frequently associated with the mesenchymal signature [12]. More recently, it has been shown that gliomas with a proneural gene expression signature displayed a distinct underlying (epi-)genetic phenotype consisting of a specific glioma-CpG island methylation phenotype (G-CIMP) related to *IDH1* mutations [19]. Gliomas harbouring *IDH1* mutations belong to the group of WHO grade II and III gliomas typically lacking vascular proliferations or account for only approximately 5% of secondary glioblastomas [20]. Therefore, the mesenchymal signature rather seems to filter out the group of primary glioblastomas which usually harbour a significantly worse prognosis as compared to its lower grade counterparts or secondary glioblastomas. In line with this assumption, the key EMT transcription factors assessed in our study were exclusively found in primary glioblastomas within the group of diffuse gliomas WHO grade II-IV. In glioblastoma, vascular proliferation is a central diagnostic hallmark separating them from lower grade astrocytomas, the latter exhibiting a significantly better prognosis [21]. Patients showing more regularly formed microvessels (“classic“ vascular pattern) show significantly longer survival times as compared to patients with vascular abnormalities in shape, size and complexity of blood vessels resulting in glomeruloid, garland-like or clustered bizarre vascular formations. This indicates that not only the number of vessels but especially the vascular morphology seems to be associated with glioma malignancy [22]. However, it is still difficult to explain how a vascular proliferation program can be associated with glioma malignancy on the one hand, but on the other being present in both the most benign WHO grade I pilocytic astrocytoma and the highest malignant glioblastoma, WHO grade IV. Of note, pilocytic astrocytomas have not been investigated for EMT-related signatures so far [7, 9]. Even though there are some differences in the vascular architecture of pilocytic astrocytomas and glioblastomas, there is a critical overlap between newly formed blood vessels of both entities comprising not only vessel immaturity or instability but also alterations in the basement membrane and pericytic coverage [23, 24]. On the one hand, human mesenchymal stem cells (hMSC) are considered to be a source of mural cells with a pericyte-like phenotype lacking endothelial cell markers [25]. In contrast, other studies claim that a large amount of glioma VAMCs or even endothelial cells derives from neoplastic glial cells with stem-like features by transdifferentiation processes [26–30]. Therefore, we also analysed glioma VAMCs, constituting the cellular source of EMT transcription factors in our cohort, for the most frequent and specific glioma-associated genetic aberrations namely *IDH1* mutation, 1p/19q LOH as well as *EGFR* amplification (Figure 3). Cells of the vascular compartment did not exhibit glioma-specific mutations. Therefore, a transition of glioma cells to VAMCs is very unlikely. These findings are strengthened by the absence of both glial and endothelial cell markers on VAMCs (Figures 2, 4). The finding that glioma VAMCs strongly express EMT transcription factors in vascular proliferations indicates that along with a pronounced morphological alteration, these non-neoplastic cells obviously undergo a mesenchymal activation process. This is further corroborated by the TCGA data analyses showing closest associated of central EMT molecules with classical VAMC markers including *CD248*, *ACTA2* and *PDGFRB* (Figure 5 and Supplementary Figure 3) [31]. Additionally, the fact that the next closest mRNA expression cluster exclusively consists of vessel-associated markers including *CD34*, *FLT1* and *KDR,* while glioma cell-related factors remain in more distanct clusters further strenghtens the findings that EMT induction is exclusively related to glioma vessels. The previously proposed descent of VAMCs from hMSCs is difficult to prove since after potential transformation of hMSCs into glioma VAMCs, the latter exclusively exhibits a classical mural cell phenotype making a distinction from resident mural cells impossible [25]. However, the possibility that a large amount of hMSCs pass the endothelial cell layer of glioma vessels subsequently dispersing between the endothelium at the inner and basement membrane at the outer side of the vascular compartment appears unlikely at first glance, but experimental data indicate that MSCs specifically graft into the glioma vasculature after intravenous injection then showing the typical marker profile of mural cells [32]. A second possibility for the increase of mural cells in both pilocytic astrocytomas and glioblastomas might be an activation and/or transformation of residual endothelial or preexisting mural cells potentially reflecting a transformation process of endothelial cells into mesenchymal cells termed endothelial-mesenchymal transition (EndoMT) [33]. Also a recruitment of endogenous brain pericytes to tumor microvasculature is a potential source of mural cells expressing EMT genes [34].

Since mural cells of pilocytic astrocytomas and glioblastoma often show an almost as highly elevated proliferation rate as compared to the glioma cells, a local activation program inducing cell division at least partly contributes to the considerably increased amount of mural cells in both brain tumor entities [24]. Since the activation of the EMT program has been considered as a predictor for glioma patient survival, we assessed the potential impact of the expression of EMT molecules in our cohort. Interestingly, we did not observe a significant association of high EMT factor levels and patient survival. On the contrary, there was even a trend for better survival of glioblastoma patients exhibiting higher TWIST levels (Supplementary Figure 5). This finding is most probably related to the fact that we stratified our cohort according to glioma subtypes meaning that lower grade gliomas with a different genetic and phenotypic profile were not analysed together with primary glioblastomas. The missing impact of EMT gene expression on patient survival is further corroborated by the fact that their strongest expression is seen in both WHO grade I astrocytomas that are mostly cured by neurosurgical resection only and WHO grade IV glioblastomas that have a very dismal prognosis. According to our findings, EMT molecules rather indicate prominent neo-angiogenesis in gliomas which is a diagnostic hallmark of WHO grade IV glioblastoma but also prominently seen in lowest grade pilocytic astrocytomas. The assumption that depletion of mural cells might impair glioma-associated angiogenesis thereby potentially inhibiting tumor growth needs further pre-clinical investigation [26].

In summary, our results show that the upregulation of central EMT effector molecules strongly reflects the amount of neo-angiogenesis in both pilocytic astrocytomas WHO grade I and glioblastomas WHO grade IV. Furthermore, we identified non-neoplastic VAMCs as unique source of expression of EMT factors in astrocytic brain tumors.

In conclusion, our findings shed a new light on the EMT program in human gliomas indicating that not neoplastic glial cells but VAMCs are the source of EMT transcription factors. Even though the expression levels of EMT molecules were not associated with patient survival in the disctinct glioma subgroups, further studies are needed to investigate the general contribution of VAMCs in gliomagenesis and progression as well as their potential role in future targeted treatment approaches.

## MATERIALS AND METHODS

### Patient data

The patient cohort consisted of 896 formalin-fixed paraffin-embedded tissue samples of 350 patients suffering from astrocytic tumors (Table 2). Tissue samples were retrieved from the archives of the Neurological Institute (Edinger Institute) of the Goethe University Frankfurt, Germany. Additionally, whole mount sections were stained, for detailed analyses, also including 5 CNS tissue samples from normal autopsy cases. The use of patient material was endorsed by the ethical committee of the Goethe University Frankfurt, Germany (GS 04/09).

**Table 2:** Patient characteristics

	Pilocytic astrocytoma, WHO°I	Diffuse astrocytoma, WHO°II	Anaplastic astrocytoma, WHO°III	Glioblastoma, WHO°IV
male/female (n)	17/30	10/6	20/15	143/109
median age (range) in years	14.0 (0-75)	30.5 (5-52)	43 (22-66)	61 (7-80)
specimens (n)	47	16	35	252
tumor localization (supratentorial/infratentorial)	16/31	16/0	34/1	250/2
median follow-up (range) in months	31.0 (0.0-142.1)	52.9 (0.0-122.1)	12.1 (0.0-164.3)	11.3 (0.0-94.0)
mIDH1(R132H) (mut/wt)	0/46	8/6	19/16	10/242

### Immunohistochemistry (single and double stainings)

Immunohistochemistry was performed on DiscoveryXT Immunohistochemistry System (Ventana, Strasbourg, France) as previously described [35]. The following anti-human antibodies were applied on 3μm-thick slides: rabbit IgG anti-SLUG (clone: C19G7, dilution 1:50, Cell Signaling, Danvers, MA, US) and mouse IgG1 anti-TWIST (clone: 2C1A, dilution 1:50, Abcam, Cambridge, UK). For double stainings the following antibodies were used: rabbit IgG anti-SLUG (clone: C19G7, dilution 1:50, Cell Signaling), mouse IgG1 anti-TWIST (clone: 2C1A, dilution 1:50, Abcam), monoclonal mouse IgG1 anti-CD31 (clone: JC70A, dilution 1:200, DakoCytomation, Glostrup, Denmark), monoclonal mouse IgG1 anti-collagen IV (clone: CIV22, dilution 1:50, DakoCytomation), monoclonal mouse IgG2a anti-alpha-SMA (clone: 1A4, dilution 1:500, DakoCytomation), polyclonal rabbit anti-GFAP (clone: Z0334, dilution: 1:500, DakoCytomation), mouse IgG2a anti-mutated IDH1 (clone: H09, dilution 1:50, dianova, Hamburg, Germany). For double immunostainings (n = 60 cases in total) first primary antibodies were applied as in single immunohistochemistry followed by heat denaturation steps, application of the second primary antibody and one drop of Universal Secondary Antibody. For visualization chromogen Fast Red was added. Finally, sections were washed, counterstained with hematoxylin and bluing reagent and mounted.

### Immunofluorescence

After deparaffinization heat pretreatment was performed in citrate buffer (pH 6). Slides were blocked with Roti-Block (Roth, Dautphetal-Buchenau, Germany) and incubated with a primary antibody mix. The first secondary antibody (Alexa Flour 488 IgG H+ L goat anti-rabbit, dilution: 1:500; clone: A11034, Invitrogen, Carlsbad, USA) was incubated for an hour. Next, specimens were labelled with the second secondary antibody (Alexa Flour 568 IgG1 goat anti-mouse, dilution: 1:500; clone: A21124, Invitrogen). Nuclear staining was performed using a mix of DAPI (4′,6-Diamidin-2-phenylindol, dilution: 1:500) and To-Pro 3 (dilution: 1:1000; Invitrogen). Endogenous fluorescence was blocked by Sudanblack (Sigma-Alderich, St. Louis, USA). All sections were washed and mounted using Fluorescent Mounting Medium (DakoCytomation). Fluorescent signals were analyzed using a C1 confocal microscope (Nikon, Japan). Further digital processing was performed using Photoshop CS3 (Adobe Systems, München, Germany).

### Silver *in-situ* hybridization (SISH)

SISH of Epidermal growth factor receptor (*EGFR)*-amplification was performed using the BenchMark XT IHC/ISH system (Ventana). After deparaffinization and pretreatment, slides were incubated with one drop of ISH-Protease followed by the *EGFR* probe. The visualization was performed with Silver C. A chromosome 7 probe (Red ISH V probe) was used as control. Afterwards the sections were washed, counterstained with hematotoxylin and bluing reagent and mounted.

### Chromogen *in-situ* hybridization (CISH)

CISH of 1p and 19q was performed by ZytoDot 2C CISH Implementation Kit (ZytoVision, Bremerhaven, Germany). After deparaffinization and blocking, pretreatment was performed with EDTA Pretreatment Solution at 95°C. After incubation with pepsine samples were rehydrated. 10μl of probes were added, fixed with Fixogum and denatured at 79°C. Hybridization was performed over night at 37°C. Next, slides were incubated with Anti-DIG and HRP/AP-Polymer. The 1p or 19q probe was visualized by AP Red Solution, the control probe by HRP-Green Solution. Slides were counterstained with Nuclear Blue followed by washing, dehydration and mounting. Loss of heterozygosity (LOH) was determined according to the guidelines of the Research Committee of the European Confederation of Neuropathological Societies [36].

### Scoring

SLUG and TWIST expression were separately assessed in vessel-associated and tumor cells taking into account staining intensity (0: no, 1: weak, 2: moderate, 3: strong) and frequency (0: 0-1%, 1: 1-10%, 2: 10-25%, 3: 25-50% and 4: >50%) of all cells showing positive nuclear staining [37]. The two results were multiplied, so that the final cell score reflected both.

### TCGA and REMBRANDT platform analyses

Gene expression signatures were analyzed in 424 primary glioblastomas and 11 normal brain samples by assessing the TCGA dataportal using Agilent 244K G4502A microarray to determine mRNA profiles (https://tcga.cancer.gov/) [38]. Subsequently, hierarchical clustering was performed by Ward’s minimum variance method. The calculations were performed using Microsoft Excel (Excel for Mac 2008; Redmond, WA, USA) and the JMP 8.0 software (SAS, Cary, NC, USA).

### Statistical analyses

The semi-quantitative SLUG and TWIST scores were assigned as ordinal scale response variables and statistical differences among WHO grades were assessed using the non-parametric Wilcoxon test followed by Bonferroni post hoc test (p < 0.05 = ^*^; p < 0.01 = ^**^; p < 0.0001 = ^***^). The association of patient survival with the response variable (SLUG or TWIST expression) was assessed by Kaplan-Meier analysis tested by log-rank and Wilcoxon test. A significance level of alpha = 0.05 was selected for all tests. Statistical analysis was performed using JMP 8.0 software (SAS, Cary, NC, USA).

## SUPPLEMENTARY MATERIALS FIGURES



## Abbreviations

Anti-DIG: Anti – Digoxigenin
αSMA: Alpha smooth muscle actin
AP: Alkaline phosphatase
CISH: Chromogen in – situ hybridization
CNS: Central nervous system
EDTA: Ethylendiamintetraacetat
EGFR: Epidermal growth factor receptor
EMT: Epithelial – mesenchymal transition
EndoMT: Endothelial – mesenchymal transition
GFAP: Glial fibrillary acidic protein
HGF: Hepatocyte growth factor
hMSC: human mesenchymal stem cells
HRP: Horseradish peroxidase
IDH – 1: Isocitrate dehydrogenase 1
LOH: Loss of heterozygosity
MET: Mesenchymal – epithelial transition
SISH: Silver in-situ hybridization
TCGA: The Cancer Genome Atlas
VAMC: vessel associated mural cells
VEGF: Vascular endothelial growth factor
WHO: World Health Organisation
