# Supplementary material for: Pericytes/vessel-associated mural cells (VAMCs) are the major source of key epithelial-mesenchymal transition (EMT) factors SLUG and TWIST in human glioma
Source: Oncotarget. 2018 May 8;9(35):24041–53. doi: 10.18632/oncotarget.25275 (PMC5963615; doi:10.18632/oncotarget.25275)
Supplement: Supplementary file 1 [file oncotarget-09-24041-s001.pdf]

Pericytes/vessel-associated mural cells (VAMCs) are the major source of key epithelial-mesenchymal transition (EMT) factors SLUG and TWIST in human glioma

SUPPLEMENTARY MATERIALS

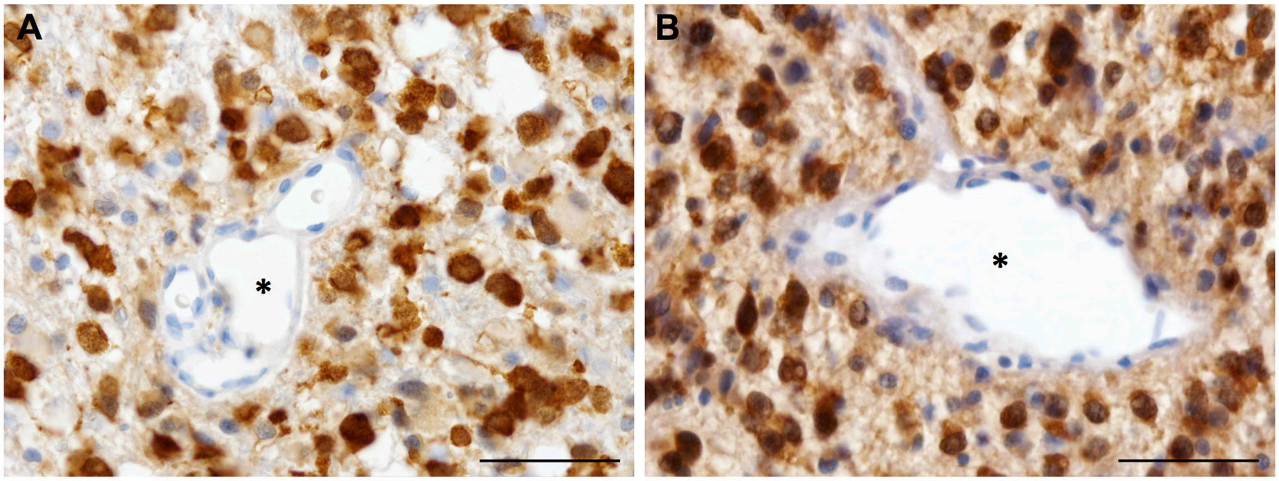

Supplementary Figure 1: Normal appearing vessels in astrocytic tumors are devoid of EMT factor expression. Double immunohistochemistry for mutated *IDH-1* (R132H) (*brown*) as marker for neoplastic glial cells in anaplastic astrocytoma WHO grade III in combination with **(A)** SLUG and **(B)** TWIST staining. Both EMT markers were absent from non-proliferating tumor-associated blood vessels (*asterisk*) in WHO grade III astrocytomas (Scale bars: A-B 50µm).

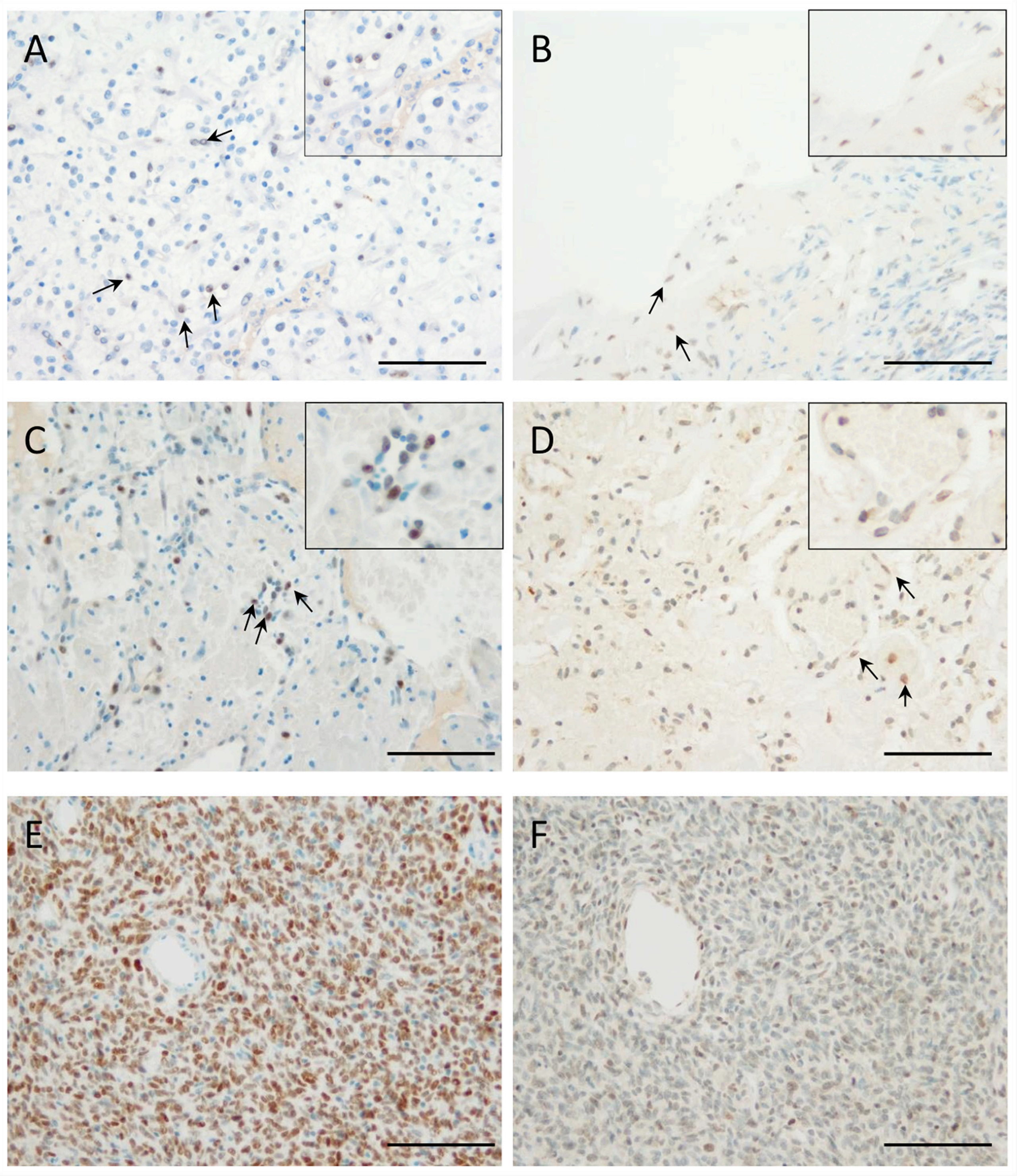

Supplementary Figure 2: SLUG and TWIST are expressed by VAMCs in vascular tumors and neoplastic cells in hemangiopericytomas. Hemangioblastoma (n=2) shows SLUG **(A)** as well as TWIST **(B)** expression associated to tumor vessels (immunohistochemistry; arrows indicating positive cells). A similarly distributed but stronger expression of SLUG **(C)** and TWIST **(D)** was seen in more malignant vascular tumors such as angiosarcomas (n=2). In hemangiopericytomas (n=2) also tumor cells show a positive staining for both SLUG **(E)** and Twist **(F)**.

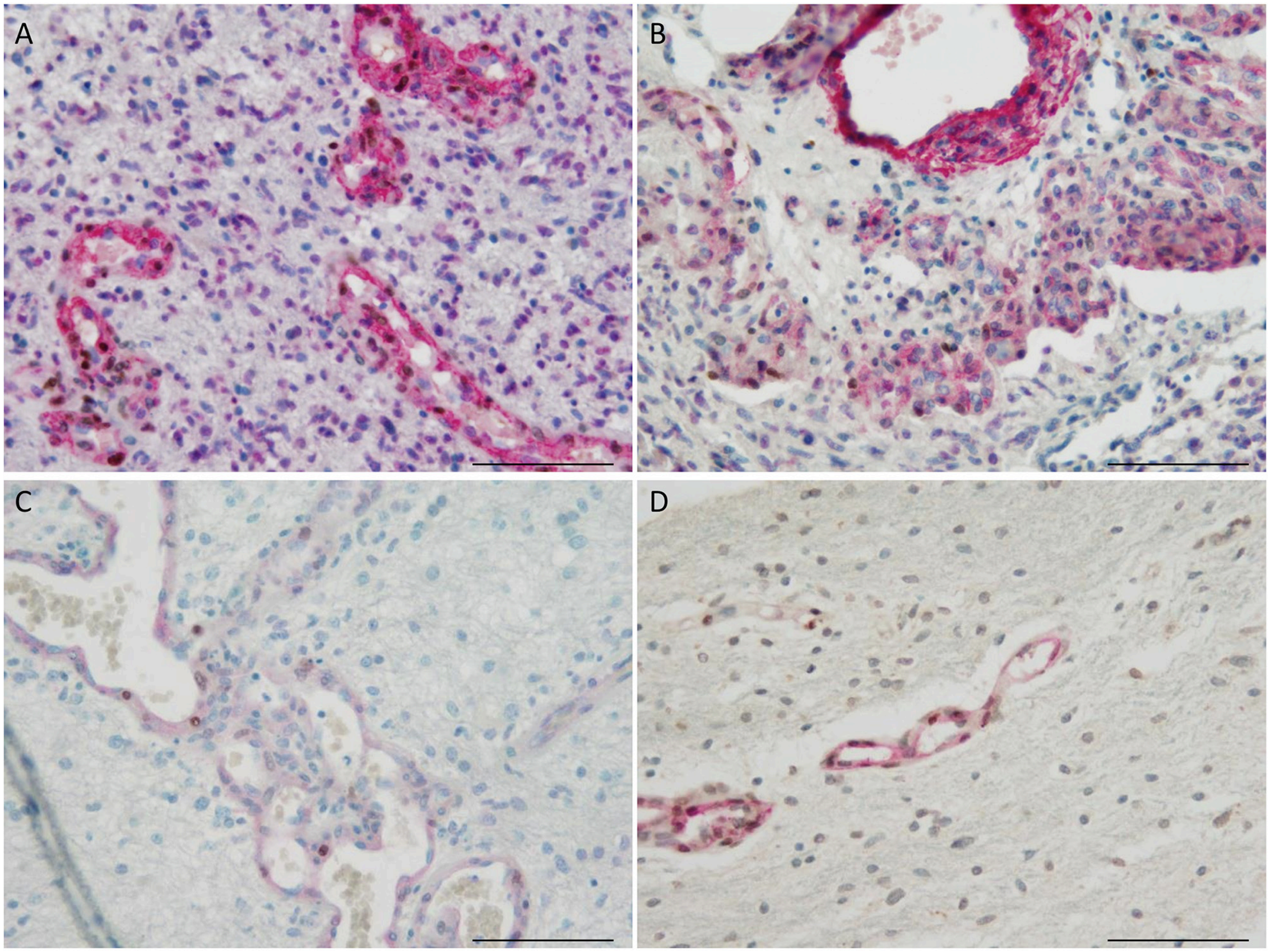

Supplementary Figure 3: SLUG and TWIST co-localize with αSMA-positive cells in human astrocytomas. Double immunohistochemistry for αSMA (*red*) and Slug (A, C: *brown*) or Twist (B, D: *brown*) in human glioblastoma **(A, B)** and pilocytic astrocytoma **(C, D)** vascular proliferations (scale bar = 100µm). The analyses reveal an unequivocal co-localisation of SLUG and TWIST (brown nuclei) and αSMA-positive cells (red).

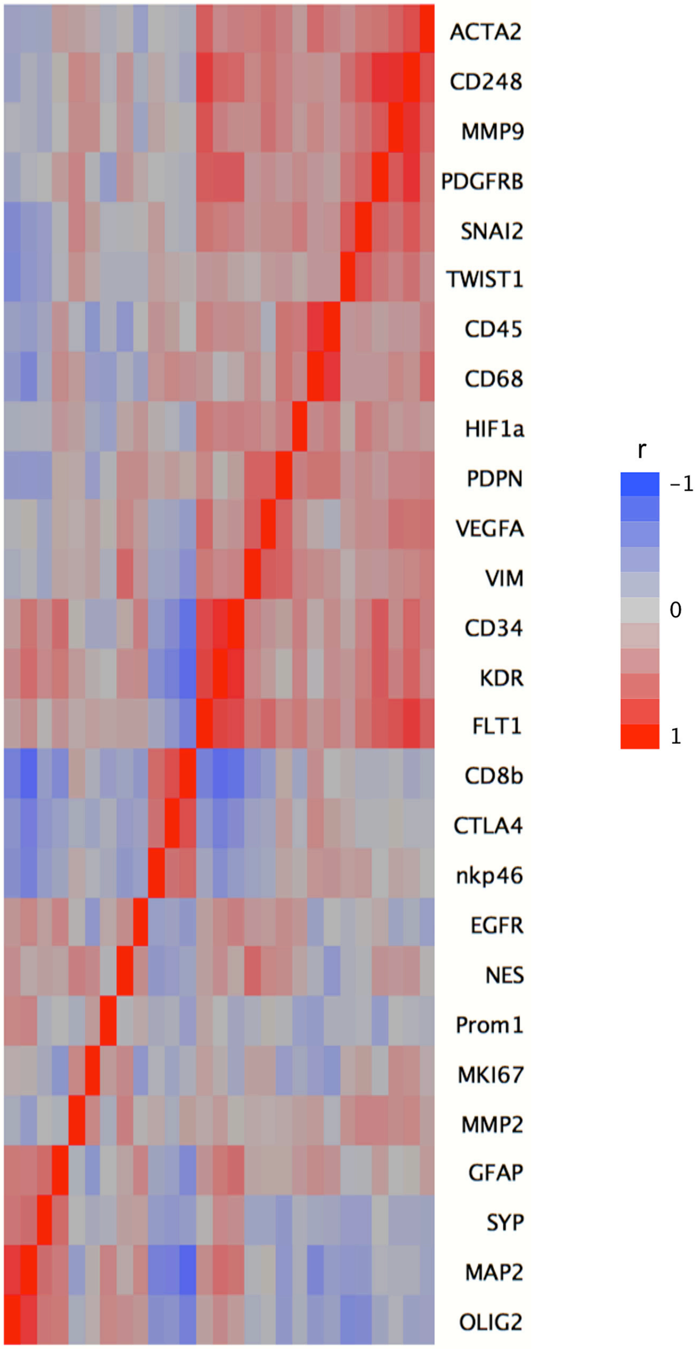

Supplementary Figure 4: Coloured correlation matrix of key factors of EMT, glioblastoma cells as well as glioblastoma micromilieu factors. This analysis contains mRNA profiles from the TCGA data portal using the Agilent 244K G4502A microarray (for further information see Figure 5). Here, we visualize the correlations between the factors already depicted in Figure 5. Dark red colour indicates a perfect positive correlation (+1) gradually decreasing to a perfect negative correlation (-1) indicated in blue. As an example, pericytic/VAMC gene expression (ACTA2, CD248, PDGFRB) shows lowest correlation with oligodendroglial or neuronal genes (OLIG2, MAP2, SYP), while strongest correlation is found with EMT (SNAI2, TWIST1) or endothelial (CD34, KDR, FLT1) genes.

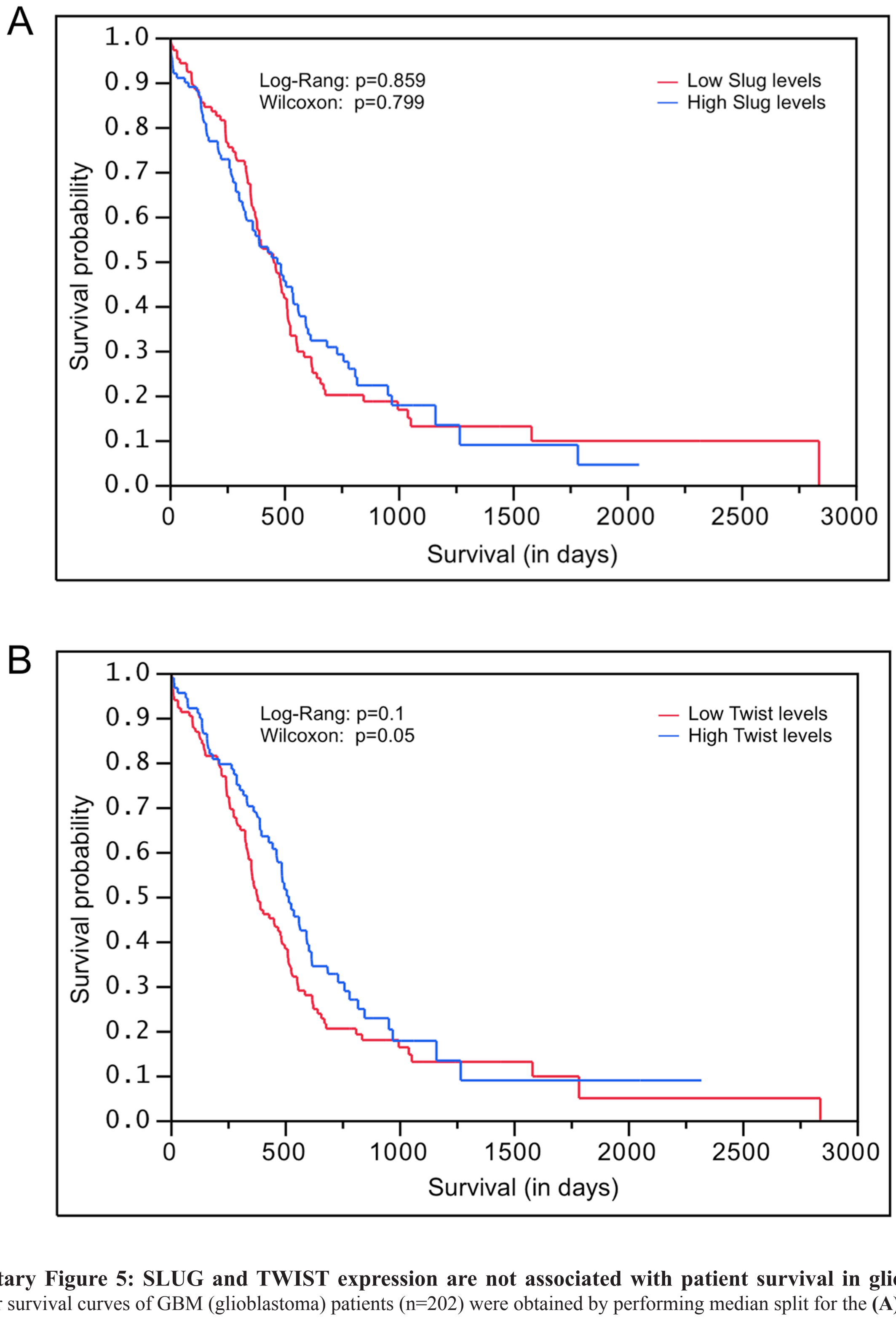

Supplementary Figure 5: SLUG and TWIST expression are not associated with patient survival in glioblastomas. Kaplan-Meier survival curves of GBM (glioblastoma) patients (n=202) were obtained by performing median split for the **(A)** SLUG (high expression > 3; low expression ≤ 3) and **(B)** TWIST (high expression >2; low expression ≤ 2) expression levels. Curves were compared by both log-rank and Wilcoxon tests; p-values for each test are indicated.
